# On the Use of Ice in Painful Conditions of the Bladder and Rectum

**Published:** 1874-09

**Authors:** Henry M. Lyman

**Affiliations:** Professor of Chemistry in Rush Medical College, and one of the Attending Physicians to the Cook County Hospital in Chicago


A MONTHLY RECORD OF

*Medicine, Surgery and the Collateral Sciences.*

EDITED BY J. ADAMS ALLEN, M.D., LL.D.; AND WALTER HAY, M.D.

VOL. XXXI.—SEPTEMBER, 1874.—No. 9.

### Original Communications.

ARTICLE I.—*On the Use of Ice in Painful Conditions of the Bladder and Rectum.* By HENRY M. LYMAN, M.D., Professor of Chemistry in Rush Medical College, and one of the Attending Physicians to the Cook County Hospital in Chicago.

Having been recently in a number of instances reminded of the value of ice in the treatment of painful affections of the rectum and bladder, I have thought it worth while to group these notes of cases, which, though presenting nothing new or particularly interesting, may serve to remind the readers of the JOURNAL of the virtues of a well-known remedy.

*Internal Piles.*—A vigorous young man, very active in his habits, suffered exceedingly with internal piles. After trying a great variety of remedies, without gaining anything more than temporary relief, the use of ice-plugs in the rectum was proposed. This measure completely subdued the pain which had tormented the patient, and after a few days of treatment he was cured. But, being of a hemorrhoidal diathesis, he is subject to occasional recurrent attacks of the disease. This, however, gives him little trouble, for the ice-plug passed above the internal sphincter muscle, soon gives him control of what was formerly a most distressing affair.

The late Dr. Chandler Gilman, of New York, used to describe to his classes the frightful suffering from piles which he had experienced when a young man, and was in the habit of employing the most fervid rhetoric to set forth the completeness of his cure by injections of ice-water into the rectum. The remedy is simple, and sometimes very effectual.

I was called in great haste, one afternoon, to see a lady who was suffering severely with intrapelvic pain. There was excessive tenesmus and urgent desire to urinate, effecting the passage of a little water from the bladder, and a great deal of slime from the rectum. I at first suspected dysentery, but the absence of inflammatory symptoms negatived this view. She had previously had a cyst in one of the broad ligaments, and the physician who saw the case with me inclined to the opinion that we had to deal with a relapse of the uterine ailment. So great was her pain that it became necessary to attempt its relief before perfecting the diagnosis. An hypodermic injection of the sulphate of morphia with atropia gave relief for two or three hours, but then the suffering was renewed. I now made a complete examination of the patient. The abdomen was flaccid, and was not painful on pressure. The uterus was in its normal position, and was not painful. Pressure against the posterior wall of the bladder gave no pain. The same was true of the lateral walls of the vagina. But when the finger was directed against the rectum, it discovered considerable swelling, great heat, and exquisite tenderness in that region. I immediately placed a large piece of ice in the vagina, and soon followed it with another. I was in a few minutes gratified by finding my patient much easier than she had been for half a day; and after introducing a vaginal suppository of morphia and belladonna, I was able to leave her perfectly comfortable. The next day she was out of bed, and has experienced no more pain.

*Irritable Bladder.*—One of my patients, not long since, during the last three weeks of pregnancy, suffered great inconvenience from pressure of the fœtal head against the bladder. Incontinence of urine was caused by this unusual pressure, and it was several weeks after delivery before the bladder recovered from the irritability thus induced. Several times the distress was sufficient to prevent sleep, in spite of the ordinary routine of opiates, diluents, suppositories, etc. I at length became aroused to a recollection of previous experience with ice, and proceeded to its use. The relief which it gave was immediate and complete.

*Vesical Irritation caused by Cantharides.*—An affectionate couple who had just retired for the night, happened to fancy that a glass of wine was all they needed to perfect their joy. Groping in the dark, the husband poured out a teacupful of wine, which he divided with his wife. The immediate effect was agreeable, but this was soon succeeded by the severest symptoms of strangury. A physician in the neighborhood was anxiously consulted, but with so little success that in the morning I was called in. The cause of the strangury had by this time been discovered in a number of Spanish flies, which had in some unaccountable manner found their way into that particular teacup which had served as a wine-glass the evening before. I advised a diligent perseverance in the use of the opiates and diluents which had been already prescribed; and I also lodged a large lump of ice in the vagina of the woman, and a smaller suppository of the same kind in the rectum of the man. They soon pronounced this *the best thing yet*, and were from that time rapidly convalescent.

*Recto-Vaginal Abscess.*—I was once requested, one hot summer evening, to see a poor woman in a distant part of the city. Arrived at her residence, I found her surrounded by a shanty full of men, women and dogs, all gesticulating wildly, and chattering like a flock of Low-Dutch magpies. With the help of two interpreters, I at length succeeded in drawing out the fact that my patient had been confined by a mid-wife about five days previous to my visit. During the course of labor she had complained that "the mid-wife hurt her." After delivery she had remained comparatively comfortable until that evening, when the mid-wife, having absorbed a quart of strong beer in honor of baby, had undertaken to inject her patient with another quart of warm soap suds. This operation caused great pain, and when taxed with malpractice, the old lady was not in a condition to deny the charge with any degree of assurance. The consequent explosion was the event which occasioned my introduction to the scene.

Thus instructed, I proceeded to an examination. My patient was a large, bony, haggard-looking creature, sweating profusely, and covered with *miliaria.* Her countenance expressed the greatest anxiety and distress. Her tongue was thickly coated with yellowish-white fur; the pulse was small, feeble, and rapid. The abdomen was overlaid with fomentations and poultices of several species. It was not distended, nor anywhere painful on pressure. The uterus was well contracted and painless. I passed two fingers into the vagina, but discovered no cause of pain until I reached the posterior wall. The rectum seemed to be greatly distended, and was very hot and exceedingly tender. Passing my forefinger into the rectum, I found nothing there, but the recto-vaginal septum was thickened, and seemed to yield under the finger like grangrenous flesh. I could detect no fluctuation, and was fearful that the septum was about to slough away. I could not quite satisfy myself with regard to the cause of this condition of the parts; but such hesitation found no place among my companions. *Scalded by a mid-wife*, was the unanimous verdict of our numerous jury, and masculine accoucheurs were at a premium in the mouth of every woman in the crowd. But something must be done, so I ordered an opiate, and advised the use of a lump of ice in the vagina. Ice! That was something used at the saloons to keep beer cool. It was an unknown commodity in that part of the city. One old crone was quite confident that it could not be a safe thing so soon after delivery. Very well. I promised to call the next morning, and hastened into the open air.

On the following day, accordingly, I found myself, with many misgivings, approaching the humble abode of my unfortunate patient. I entered. The cottage was clean and quiet. Its only occupants were the sick woman, her baby, and a single attendant. She was sitting in a rocking-chair, and a smile of gratitude rippled from ear to ear across her rugged features. "Was she better?" "Yah!"

The powders had done her no good (?); so, after a grand family debate, it had been decided to obey the doctor, and her husband had sallied forth into the night in search of ice. A benevolent butcher had been prevailed upon to furnish the article, and, sometime after midnight, the first suppository of ice was duly placed in position. Speedy relief was the result, and my patient slept till morning. Her symptoms all indicated great improvement, and, during the whole process of suppuration, until the evacuation of an abscess through the rectum, a week afterwards, a lump of ice in the vagina was always sufficient to prevent the recurrence of severe pain.

